# Staging FDG PET-CT changes management in patients with gastric adenocarcinoma who are eligible for radical treatment

**DOI:** 10.1007/s00259-019-04429-x

**Published:** 2019-08-03

**Authors:** Karen D. Bosch, Sugama Chicklore, Gary J. Cook, Andrew R. Davies, Mark Kelly, James A. Gossage, Cara R. Baker

**Affiliations:** 1 Department of Upper GI Surgery, Guy’s & St Thomas’ Hospital, London, SE1 7EH UK; 2 Department of Cancer Imaging, School of Biomedical Engineering and Imaging Sciences, King’s College London, St Thomas’ Hospital, London, SE1 7EH UK; 3 King’s College London and Guy’s & St Thomas’ PET Centre, St Thomas’ Hospital, London, SE1 7EH UK

**Keywords:** Gastric cancer, Cancer staging, PET-CT, Metastases

## Abstract

**Aim:**

18-fluorodeoxyglucose positron emission tomography-computed tomography (FDG PET-CT) is valuable in the management of patients with oesophageal cancer, but a role in gastric cancer staging is debated. Our aim was to review the role of FDG PET-CT in a large gastric cancer cohort in a tertiary UK centre.

**Methods:**

We retrospectively reviewed data from 330 patients presenting with gastric adenocarcinoma between March 2014 and December 2016 of whom 105 underwent pre-treatment staging FDG PET-CT scans. FDG PET-CT scans were graded qualitatively and quantitatively (SUV_max_) and compared with staging diagnostic CT and operative pathology results (*n* = 30) in those undergoing resection.

**Results:**

Of the 105 patients (74 M, median age 73 years) 86% of primary tumours were metabolically active (uptake greater than normal stomach) on FDG PET-CT [41/44 (93%) of the intestinal histological subtype (SUV_max_ 14.1 ± 1.3) compared to 36/46 (78%) of non-intestinal types (SUV_max_ 9.0 ± 0.9), *p* = 0.005]. FDG PET-CT upstaged nodal or metastastic staging of 20 patients (19%; 13 intestinal, 6 non-intestinal, 1 not reported), with 17 showing distant metastases not evident on other imaging. On histological analysis, available in 30 patients, FDG PET-CT showed low sensitivity (40%) but higher specificity (73%) for nodal involvement.

**Conclusion:**

FDG PET-CT provides new information in a clinically useful proportion of patients, which leads to changes in treatment strategy, most frequently by detecting previously unidentified metastases, particularly in those with intestinal-type tumours.

## Introduction

Gastric adenocarcinoma can have a poor prognosis, especially in Western countries where presentation is often at a more advanced stage at the time of diagnosis [1]. Those with early tumours, with no or limited nodal involvement, may have surgical resection combined with either neoadjuvant or adjuvant chemotherapy, whereas patients with metastases (M1) are typically managed by palliative means, including chemotherapy and best supportive care without resection. Therefore, it is important to identify appropriate surgical candidates by enhancing the accuracy of pre-treatment staging, which is the basis for treatment decisions [2]. A further consideration is the potential morbidity and mortality from gastric surgery [3], mandating accurate selection of patients who will benefit from surgery and are not harbouring undetected metastatic disease, which may render surgical intervention futile.

Staging of gastric cancer is typically based on endoscopic biopsy, computed tomography (CT) and laparoscopy for those deemed to have loco-regional disease. Positron emission tomography (PET)-CT combines anatomic and functional information to allow diagnosis and staging of cancer. PET-CT with 18-fluoro-2-deoxy-D-glucose (FDG) tracer is commonly used in oncology and has now become more widely available [4, 5].

In upper gastrointestinal cancer, FDG PET-CT has been shown to be valuable in patients with oesophageal cancer due to its high diagnostic sensitivity for the primary tumour [6], additional staging information regarding nodal status [7] and detection of distant metastases [8–11] thus identifying those patients with occult metastatic disease, or more advanced locoregional node involvement that would benefit from neo-adjuvant chemotherapy.

The usefulness of FDG PET-CT in preoperative staging of gastric adenocarcinoma in particular has been debated, with wide estimates of published sensitivity for detecting the primary tumour ranging from 60 to 94% [11–15]. In addition, physiological FDG uptake in the stomach can be variable and a malignant focus of disease may be obscured by physiological or benign pathological uptake in the stomach wall, such as acute gastritis [16, 17]. Differences in FDG uptake between the histological subtypes have also been reported, with higher uptake in cancers of intestinal type and more advanced tumours [18]. Reports have indicated low rates of detection in more distal tumours, early (T1, T2) tumours [19] and those of diffuse histological subtype [20]. It is therefore not routinely recommended in the UK, according to recent guidelines [21].

As FDG PET-CT has become more routinely available, more studies have examined its role in the staging of gastric adenocarcinoma. Recent reports have estimated the added diagnostic value of FDG PET-CT for detecting occult metastases as 6–10% [11, 15, 22, 23] with accompanying reductions in surgical morbidity, mortality and cost by the avoidance of unnecessary gastrectomy. Due to relatively low spatial resolution, FDG PET-CT does not add to assessment of primary tumour (T) stage [24]. For the assessment of locoregional lymph nodal staging in gastric cancer, FDG PET-CT is more specific but less sensitive than CT alone [25–27]. However, recent studies have shown poorer prognosis for patients with FDG-avid nodes [11, 28, 29], suggesting an additional role for PET-CT in identifying patients at higher risk of metastasis.

In the present retrospective study, we aimed to investigate the usefulness of PET-CT in the staging work-up of patients with gastric adenocarcinoma and whether this has an impact on subsequent management.

## Methods

### Patient population

The records of patients referred for discussion at the Upper Gastrointestinal Multidisciplinary Team (MDT) meeting at Guy’s and St Thomas’ Hospital NHS Foundation Trust between March 2014 and December 2016 were analysed. All patients with a new diagnosis of biopsy-proven gastric adenocarcinoma were identified. Patients with Siewert Type I or II gastro-oesophageal tumours were excluded from the study; Type III tumours were included. We collected demographic data, tumour characteristics, radiographic and FDG PET-CT scan details, and treatment information from the patients’ electronic record. TNM stage, as per MDT consensus, was recorded both pre- and post-PET-CT scan. The contemporary 7th Edition of the American Joint Committee on Cancer (AJCC) manual of staging criteria was used to further classify staging information [2]. For those patients who subsequently underwent surgical resection the type of resection, number of lymph nodes resected and number of nodes positive for metastatic adenocarcinoma were recorded.

### FDG PET-CT grading

18-FDG-PET-CT scans were performed using the standard clinical oncology protocol at a single high-volume institution with double reporting as standard procedure. Scans were acquired 90 min after intravenous injection of 350 MBq FDG (range 283 MBq to 389 MBq). Images were acquired from skull base to upper thighs with 3 min per bed position using a GE Discovery 710 PET/CT scanner (GE Healthcare, Chicago, USA). A low-dose CT scan (140 kV, 10 mA, 0.5 s rotation time, and 40 mm collimation) was performed at the start of imaging to provide attenuation correction and an anatomical reference. PET images were reconstructed with a time-of-flight ordered subset expectation maximisation algorithm (2 iterations, 24 subsets) with a reconstructed slice thickness of 3.27 mm and pixel size 4.7 mm. FDG PET-CT scan reports were analysed and grouped as follows:

- 1 = no uptake in primary
- 2 = increased uptake in primary compared to physiological gastric activity only
- 3 = increased uptake in primary and local nodes
- 4 = increased uptake in primary ± nodes and distant metastases

Group 4 includes any positivity that would indicate metastatic disease (and therefore lead to M1 staging), which includes both solid organ and distant lymph node disease [2]. In addition, a record was kept of whether FDG uptake unrelated to the gastric primary was described in the PET-CT report, and whether this then led directly to additional investigations. For quantitative analysis, a single, experienced nuclear medicine physician used a dedicated work-station to analyse FDG PET data for the maximum standardised uptake value (SUV_max_).

### Change in staging following FDG PET-CT

For each patient who had TNM staging information available both pre- and post-FDG PET-CT, the effect of the additional scan on the staging was grouped as follows:

- A = negative scan
- B = no change in stage
- C = up-stage N
- D = up-stage M

Group C indicates local nodes identified and involved on FDG PET-CT (i.e. N0 up-staged to N1, N2 or N3) and group D, newly diagnosed metastases (i.e. M0 up-staged to M1).

### Statistics

To test for statistical differences between mean values, unpaired Student’s T test was used. *P*-values below 0.05 were considered statistically significant.

## Results

### Patient and tumour characteristics

A total of 330 patients with newly diagnosed, biopsy-proven gastric adenocarcinoma were identified between March 2014 and December 2016. The majority of patients were male (65%, ratio 1.9:1) and median age at diagnosis was 73 years (range, 24–95 years). Histological analysis after endoscopic biopsy showed Lauren classifications consisting of 41% intestinal, 23% diffuse, 11% mixed and 24% not reported. Tumour differentiation at biopsy was 56% poorly differentiated, 33% moderately differentiated 2% well differentiated and 8% not reported. At the time of initial MDT discussion, following endoscopic biopsy and staging CT, 31% patients were found to have metastatic disease. These results are summarised in Table 1.

**Table 1:** Overview of patient and tumour characteristics

Characteristic		Overall, N = 330, *n* (%)	PET-CT group, N = 105, *n* (%)
Sex	Male Female	216 (65)	74 (70)
		114 (35)	31 (30)
Median age		73 (range 24–95)	69 (range 24–87)
Lauren’s classification	Intestinal Diffuse Mixed Other Not reported	135 (41) 77 (23) 37 (11) 3 (1) 78 (24)	44 (42) 30 (29) 16 (15) 0 (0) 15 (14)
Differentiation	Well Moderate Poor Not reported	8 (2) 110 (33) 186 (56) 26 (8)	1 (1) 36 (34) 64 (61) 4 (4)
Metastases	M0 M1	227 (69) 103 (31)	75 (71) 30 (29)

### Uptake distribution of FDG

A total of 105 patients (32%) underwent PET-CT as part of the staging process. Breakdown of the patient and tumour characteristics of this sub-group of patients is also shown in Table 1. FDG PET-CT scans were not performed in the remainder of cases due to findings of metastatic disease on CT 88 (27%), frailty and limited treatment decisions 56 (17%), early/straight to surgery 45 (14%), patient declined 2 (1%) and not documented 34 (9%).

**Fig. 1:**
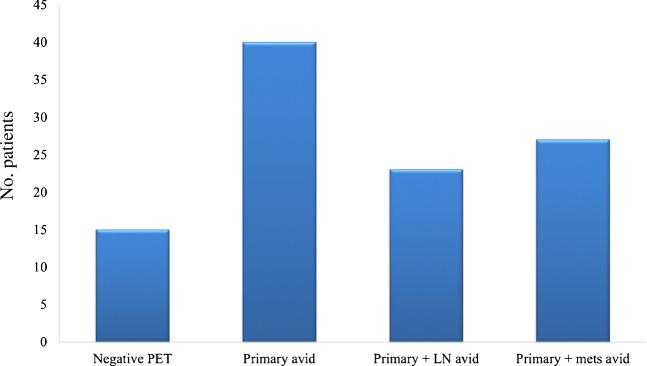
Uptake distribution of FDG (*n* = 105)

**Fig. 2:**
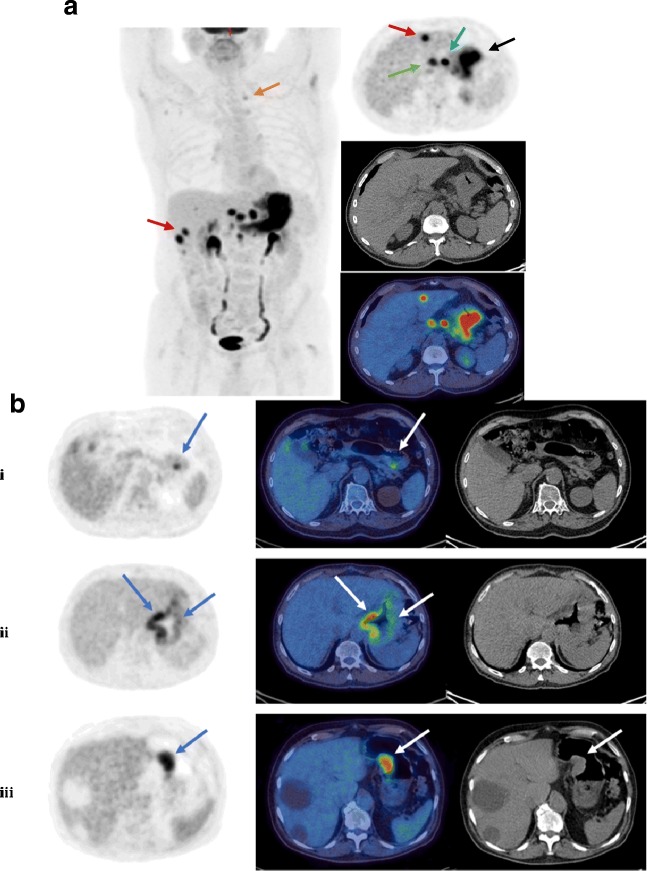
Example FDG PET-CT images. **a***Left*: maximum intensity projection (MIP) showing diffuse gastric malignancy with multiple left gastric, coeliac, portal and retroperitoneal nodes, bilobar liver metastases and a metastatic superior mediastinal node. *Right*: axial (PET – *top*, CT – *middle*, fused PET-CT – *bottom*). *Arrows* indicate the gastric primary (*black*), a regional left gastric node (*blue*), bilobar liver metastases (*red*), a portal node (*green*) and a small left superior mediastinal node (*orange; left image*). **b** Example FDG PET-CT images from three patients with different uptake distribution. Shown are axial images, from left to right: MIP, fused PET-CT, CT only; (i) Focal primary tumour uptake, lesser curvature of stomach, (ii) Diffuse primary tumour uptake along both greater and lesser curvatures of stomach, (iii) Lesion along lesser curvature of stomach corresponding to soft tissue on CT. Note photopaenic large simple hepatic cysts

Analysis of FDG PET-CT scan reports showed that 86% of patients had an FDG-avid primary tumour (groups 2–4; 90 of 105 PET-CT scans), with breakdown of distribution as follows: 40 (38%) patients had a positive primary tumour only (group 2), 23 (22%) had positive primary and locoregional lymph nodes (group 3), and 27 (26%) had positive primary and distant metastases (group 4) (Fig. 1). Example images are shown in Fig. 2. The average size of FDG negative tumours was 39.7 mm (range 10–80 mm), i.e. tumours were larger than the resolution of PET-CT. The presence of FDG uptake unrelated to the gastric primary was also recorded and found to be present in 71 (68%) of FDG PET-CT scan reports. Thirteen of 105 (12%) patients required further investigation of incidental findings based on FDG PET-CT results. These included endobronchial ultrasound (EBUS) for upper mediastinal nodes (*n* = 4), colonoscopy (*n* = 2), cystoscopy (n = 2), fine needle aspiration of supraclavicular node (*n* = 1), breast ultrasound (n = 1) and pleural biopsy (n = 1). Incidental uptake typically represented distant inflammatory processes. However, in two cases a synchronous primary tumour was newly identified elsewhere (squamous cell carcinoma of the lung and B cell lymphoma of cervical lymph nodes).

**Fig. 3:**
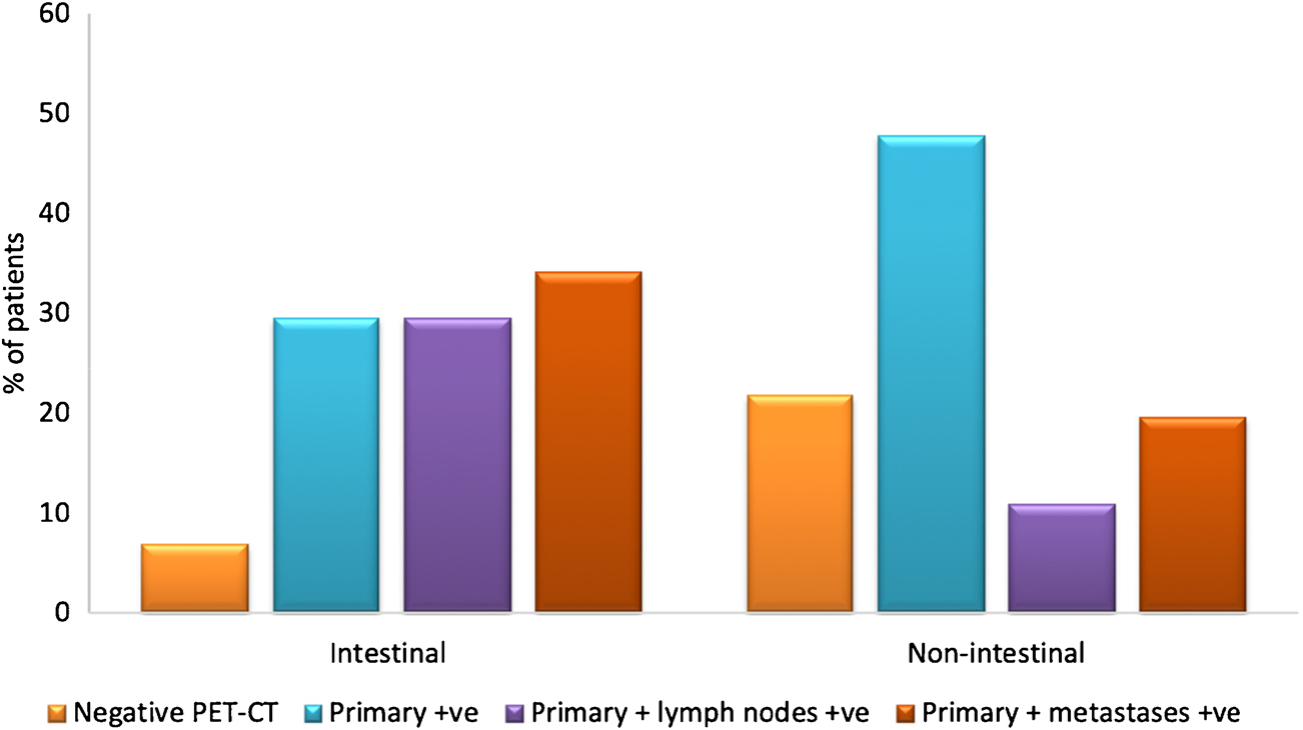
Uptake distribution of FDG by histological subtype (*n* = 90). Percentage of patients for intestinal and non-intestinal subtypes, respectively: negative: FDG PET-CT 7% (3/44), 22% (10/46); primary +ve: 30% (13/44), 48% (22/46); primary +ve and lymph nodes +ve: 30% (13/44), 11% (5/46); primary +ve and metastases +ve: 34% (15/44), 20% (9/46)

Of the 90 FDG positive primary tumours, those of intestinal subtype were more likely to have FDG-avid lymph nodes (*n* = 13/44, 30%) or FDG-avid metastases (*n* = 15/44, 34%) compared with non-intestinal-type tumours (*n* = 5/46, 11% and *n* = 9/46, 20%; lymph node and metastasis positivity, respectively; Fig. 3).

### FDG-avidity of the primary tumour

Semi-quantitative analysis of FDG-avidity was obtained by measuring the SUV_max_ of the primary tumour. From this analysis, the primary tumour was found to have a mean SUV_max_ of 12.0 ± 0.9 (mean ± standard error of the mean (SEM); range, 2.6–41.4). Intestinal-type primary tumours were PET-positive in 93% (41/44) of cases, compared with 36/46 (78%) non-intestinal-type tumours (diffuse, mixed). In the remaining 15 cases, histological subtype was not recorded. In addition, mean SUV_max_ uptake was significantly higher in tumours of intestinal vs. non-intestinal type tumours (14.1 ± 1.3 vs. 9.0 ± 0.9; mean ± SEM, *p* = 0.005). Primary tumours that had metastasised also had higher SUV_max_ values (11.2 ± 1.2 for M0 disease vs. 14.6 ± 1.7 for M0; mean ± SEM; *p* = 0.052).

### Changes in staging following FDG PET-CT

**Fig. 4:**
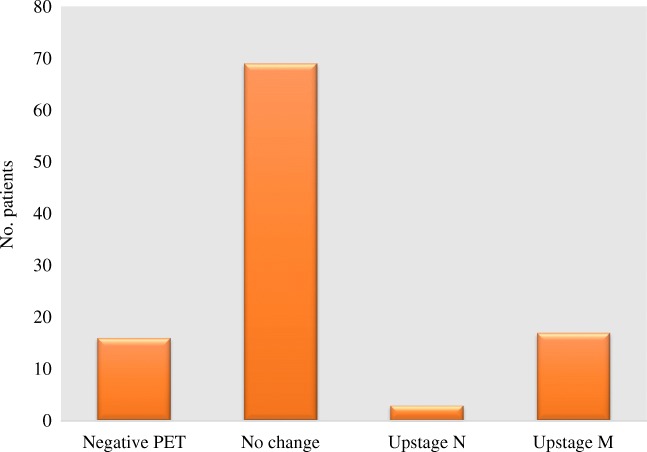
Change in TNM stage following PET-CT (*n* = 105). Negative PET = 16 patients; no change = 69 patients; upstage N = 3 patients; upstage M = 17 patients

**Fig. 5:**
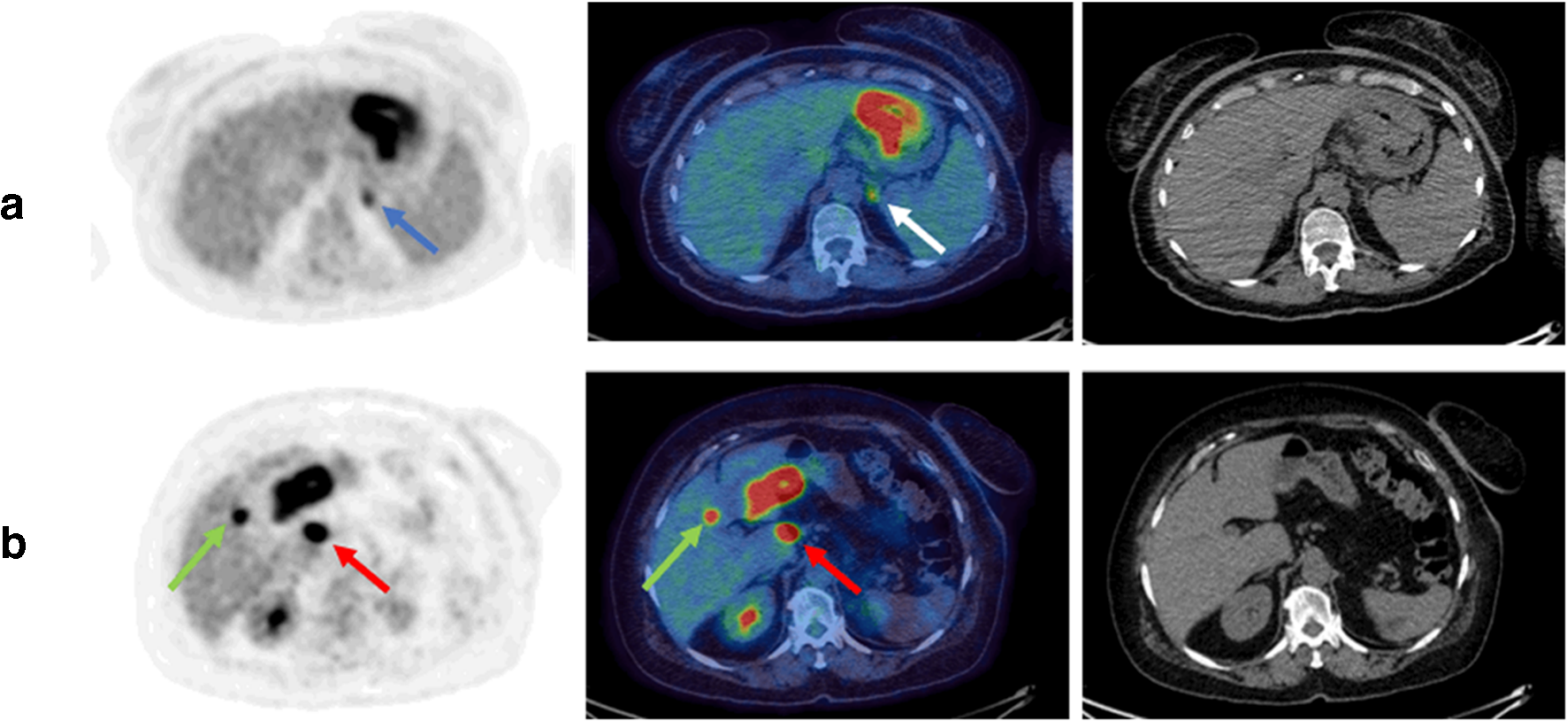
Example FDG PET-CT images from patients with occult metastases. Shown are axial images, from left to right: MIP, fused PET-CT, CT only; (a) positive gastric primary plus occult adrenal metastasis (*blue and white arrows*), (b) positive gastric primary with porta hepatis node (*red arrows*) plus occult hepatic metastasis (*green arrows*)

TNM stage prior to and following FDG PET-CT was recorded for each patient where this information was available (105 patients) and the impact of the scan on the staging was grouped (see *Methods* section, above). New information from FDG PET-CT lead to up-staging in 20/105 patients (groups C and D; 19%) by demonstration of previously undetected positive lymph nodes or metastases (Fig. 4). Of those patients who were up-staged, the majority was due to newly detected metastases (group D; 17 patients, 16%). These newly detected occult metastases included distant nodes (8 patients), peritoneal nodules (6 patients), liver metastases (2 patients) and adrenal metastasis (1 patient). Example images from patients with occult metastases are shown in Fig. 5. In 85 patients (groups A and B; 81%), no new information was provided as the FDG PET-CT was either negative or in agreement with the staging after initial MDT discussion (i.e. staging prior to FDG PET-CT).

### Concordance between PET-CT and histological lymph node status

From a total of 330 patients with newly diagnosed gastric adenocarcinoma, 54 (16%) underwent resectional surgery. Of these patients, 30 had a pre-surgical FDG PET-CT scan to enable comparison of pre-operative regional lymph node FDG-positivity and histological nodal status. Seventeen patients’ nodal status was concordant on FDG PET-CT and histological analysis (Table 2; six FDG and histology positive, 11 FDG and histology negative). Discordance between pre-treatment FDG PET-CT and histological nodal status was seen in 13 patients (Table 2; 4 FDG positive, histology negative, 9 FDG negative, histology positive). Of this discordant group, the majority of those with positive lymph nodes not predicted by PET-CT (i.e. FDG negative, histology positive) were of non-intestinal subtype (78%). In the concordant groups, the mix of intestinal and non-intestinal histological subtypes were evenly distributed. This suggests that nodal positivity is predicted more accurately in intestinal than non-intestinal tumours. These data correspond to an overall sensitivity and specificity of PET-CT nodal detection of 40% and 73%, respectively.

**Table 2:** Concordance between lymph node status on FDG PET-CT and histological analysis

Lymph node status	Histology positive	Histology negative
FDG positive	6	4
FDG negative	9	11

Nineteen of 30 patients underwent neo-adjuvant chemotherapy. Of these, discordance between pre-treatment FDG PET-CT and histological nodal status was seen in seven patients (2 FDG positive, histology negative; 5 FDG negative, histology positive). Four out of five patients in the neo-adjuvant group with positive lymph nodes not predicted by PET-CT were of non-intestinal subtype. Eleven patients proceeded directly to surgery following FDG PET-CT without neo-adjuvant treatment. In two cases, chemotherapy was contraindicated due to gastric outlet obstruction and cardiac comorbidity. In the remaining nine patients, pre-operatively staged as early, six of nine were finally staged as T1/2 (N0–2) and in three cases T3/4 but N0.

## Discussion

Here we report the results of a large retrospective study evaluating the usefulness of FDG PET-CT in pre-operative staging of gastric cancer. The population is urban and screening programs are not in use. We describe a cohort of predominantly older patients, with twice as many males as females, consistent with previous reports [30, 31]. Tumours were mostly poorly differentiated and a substantial proportion (31%) of patients were found to have metastatic disease at diagnosis.

In order to identify those who are likely to benefit from curative surgery, accurate staging information is essential. The role of FDG PET-CT as part of the staging algorithm in gastric cancer has been debated. This study confirms that it does have a place in modern gastric staging and complements other imaging modalities. We report an additional 16% of patients in whom occult metastases, consisting of distant lymph nodes or solid organ disease, were identified solely on FDG PET-CT, thus rendering those patients unsuitable for curative surgery. This guides treatment strategies away from the morbidity and mortality associated with a futile surgery, with no benefit for long-term survival, and avoidance of the considerable associated cost. This is consistent with previous studies, which have reported added information regarding occult metastases in up to 10% of patients [11, 15, 22, 23]. A possible reason for the higher detection rate of metastases in our study compared to previous reports include improved scanning technology (e.g. availability of time of flight PET acquisition), resulting in improved sensitivity.

In agreement with previous reports, sensitivity for the primary tumour is greater for the intestinal histological subtype. In addition, we found that tumours of the intestinal type have greater rates of FDG-avid lymph nodal and metastatic positivity. Indeed, of the patients up-staged by new information from FDG PET-CT, more than twice as many were of the intestinal subtype. It is likely that FDG PET-CT is more sensitive to small deposits of intestinal versus non-intestinal-type tumours. This hypothesis is supported by our results, and others, of higher SUV_max_ values reflecting this pattern [11], and superior prediction of lymph node metastasis for intestinal tumours when pre-surgical FDG PET-CT is compared with histological analysis. Interestingly, Findlay and colleagues [11] did not detect a difference in the rate of primary tumour avidity between the histological subtypes, despite agreement with our findings on subtype differences in SUV_max_.

Lymph nodal status on pre-surgical staging guides oncological and surgical strategy. Several studies have shown limited sensitivity of FDG PET-CT in evaluating lymph node metastases in gastric cancer [32–34], with values for sensitivity typically less than 50%, and the results of the present study are in agreement with these findings. It has been suggested that spatial resolution of FDG PET-CT is not high enough to differentiate the primary tumour from positive perigastric nodes [33], in addition to the frequent finding of nodal metastases in nodes that are not enlarged by CT criteria, i.e. subcentimetre. Our reported specificity in lymph nodal status is much higher than the sensitivity, 73%, although this figure is lower than that reported by others [33, 29]; this was possibly due to patients in our surgical cohort who underwent neo-adjuvant chemotherapy and downstaging. Interestingly, recent studies specifically examining the significance of nodal FDG avidity have found poorer prognosis among patients with nodal FDG positivity, suggesting that PET-CT may identify a subset of cancers with a greater propensity to metastasise [11, 29].

A number of factors complicating the usefulness of FDG PET-CT in gastric cancer staging have been identified in the present study. One example is that a significant proportion of tumours do not take up the tracer (14%), so no staging information is added in these patients. This is not a function of smaller tumour size as the average size of 18F-FDG negative tumours is close to 4 cm, with the smallest tumour measuring 1 cm. Additionally, physiological or inflammatory uptake in non-malignant gastric mucosa, for example, induced by *Helicobacter pylori* infection, can obscure a gastric cancer and provide difficulty with primary tumour identification [35]. Furthermore, more than half of the FDG PET-CT scans reported incidental findings, leading to additional tests and MDT discussion, which resulted in delays to treatment. The incidental findings were usually benign, distant inflammatory processes. However, in two cases a synchronous unrelated primary tumour was identified.

Limitations of the study include its retrospective nature, which means there is a risk that the cohort of patients undergoing additional staging investigations, such as FDG PET-CT, may exhibit a degree of referral bias, potentially overestimating the usefulness of the scans. Comparison of scan and histological results must also be interpreted carefully in view of some patients undergoing neo-adjuvant therapy, therefore potentially changing the histological results and increasing the time interval between staging and histological analysis. Finally, FDG PET-CT scans were initially reported by multiple team members so there is the possibility of inter-reporter variation. However, this risk is mitigated by the routine practice of double-reporting of scans and a highly experienced team. Additionally, here we reflect real world results rather than controlled experimental conditions.

In conclusion, this study confirms that selected use of FDG PET-CT scanning has a place in the modern staging algorithm of gastric cancer in the UK. Specifically, those patients with no obvious metastases on initial staging, who are eligible for radical curative therapy, would benefit from additional staging information. The most useful aspect is the detection of occult metastases, allowing appropriate identification of surgical candidates and avoiding futile surgery in those with M1 disease. Although valuable information was elucidated in all histological subtypes, FDG PET-CT is particularly sensitive to those tumours of intestinal type. Future, prospective large studies are needed to further investigate the role of FDG PET-CT in N staging and also in the post-treatment phase, such as in the monitoring of response to chemotherapy and prognostic assessment.
